# Cysteamine Suppresses Invasion, Metastasis and Prolongs Survival by Inhibiting Matrix Metalloproteinases in a Mouse Model of Human Pancreatic Cancer

**DOI:** 10.1371/journal.pone.0034437

**Published:** 2012-04-20

**Authors:** Toshio Fujisawa, Benjamin Rubin, Akiko Suzuki, Prabhudas S. Patel, William A. Gahl, Bharat H. Joshi, Raj K. Puri

**Affiliations:** 1 Tumor Vaccines and Biotechnology Branch, Division of Cellular and Gene Therapies, Center for Biologics Evaluation and Research, Food and Drug Administration, Bethesda, Maryland, United States of America; 2 Department of Ophthalmology, Suburban Hospital, Johns Hopkins School of Medicine, Bethesda, Maryland, United States of America; 3 Section on Human Biochemical Genetics, Medical Genetics Branch, National Human Genome Research Institute, National Institutes of Health, Bethesda, Maryland, United States of America; National Cancer Center, Japan

## Abstract

**Background:**

Cysteamine, an anti-oxidant aminothiol, is the treatment of choice for nephropathic cystinosis, a rare lysosomal storage disease. Cysteamine is a chemo-sensitization and radioprotection agent and its antitumor effects have been investigated in various tumor cell lines and chemical induced carcinogenesis. Here, we investigated whether cysteamine has anti-tumor and anti-metastatic effects in transplantable human pancreatic cancer, an aggressive metastatic disease.

**Methodology/Principal Findings:**

Cysteamine's anti-invasion effects were studied by matrigel invasion and cell migration assays in 10 pancreatic cancer cell lines. To study mechanism of action, we examined cell viability and matrix metalloproteinases (MMPs) activity in the cysteamine-treated cells. We also examined cysteamine's anti-metastasis effect in two orthotopic murine models of human pancreatic cancer by measuring peritoneal metastasis and survival of animals. Cysteamine inhibited both migration and invasion of all ten pancreatic cancer cell lines at concentrations (<25 mM) that caused no toxicity to cells. It significantly decreased MMPs activity (*IC*_50_ 38–460 µM) and zymographic gelatinase activity in a dose dependent manner *in vitro* and *in vivo*; while mRNA and protein levels of MMP-9, MMP-12 and MMP-14 were slightly increased using the highest cysteamine concentration. *In vivo*, cysteamine significantly decreased metastasis in two established pancreatic tumor models, although it did not affect the size of primary tumors. Additionally, cysteamine prolonged survival of mice in a dose-dependent manner without causing any toxicity. Similar to the *in vitro* results, MMP activity was significantly decreased in animal tumors treated with cysteamine. Cysteamine had no clinical or preclinical adverse effects in the host even at the highest dose.

**Conclusions/Significance:**

Our results suggest that cysteamine, an agent with a proven safety profile, may be useful for inhibition of metastasis and prolonging the survival of a host with pancreatic cancer.

## Introduction

Cysteamine is a simple aminothiol and anti-oxidant that has potential for the treatment of radiation sickness, neurological disorders and cancer. Cysteamine is also approved by the Food and Drug Administration for the treatment of patients with cystinosis, an autosomal recessive disorder characterized by accumulation of cystine in lysosomes [1], [2]. The pharmacokinetics and adverse effects of cysteamine have been very well studied [3]–[5]. In the cancer field, many studies have reported anti-cancer effects of cysteamine with respect to cancer development and proliferation. Cysteamine prevented not only the development of metaplasia but also carcinogenesis of mammary tumor and gastric cancers induced chemically and by radiation [6]–[8]. Cysteamine by itself or conjugated with nanoparticles or other compounds suppress cancer cell proliferation derived from neural neoplastic tumors [9], SMMC-7721 hepatocellular carcinoma [10], breast cancer [11], and melanoma cell lines [12] *in vitro*. These derivatives seem to inhibit the growth of cancer cells, synergize the effect of other anti-cancer drugs, induce apoptosis or exert cytotoxic effects on DNA replication. Cysteamine is also found to exert a biphasic effect on the autophagy by inducing the formation of autophagosomes at early time points but autophagic degradation at later time points [13]. Cysteamine is known to inhibit intestinal neoplasia induced by X-irradiation [6] and gastric carcinogenesis induced by N-methyl-N'-nitrosoguanidine in rats [9], [14]. However, there is no report in the literature addressing the anti-invasive or anti-metastatic effects of cysteamine in human cancers.

**Figure 1:**
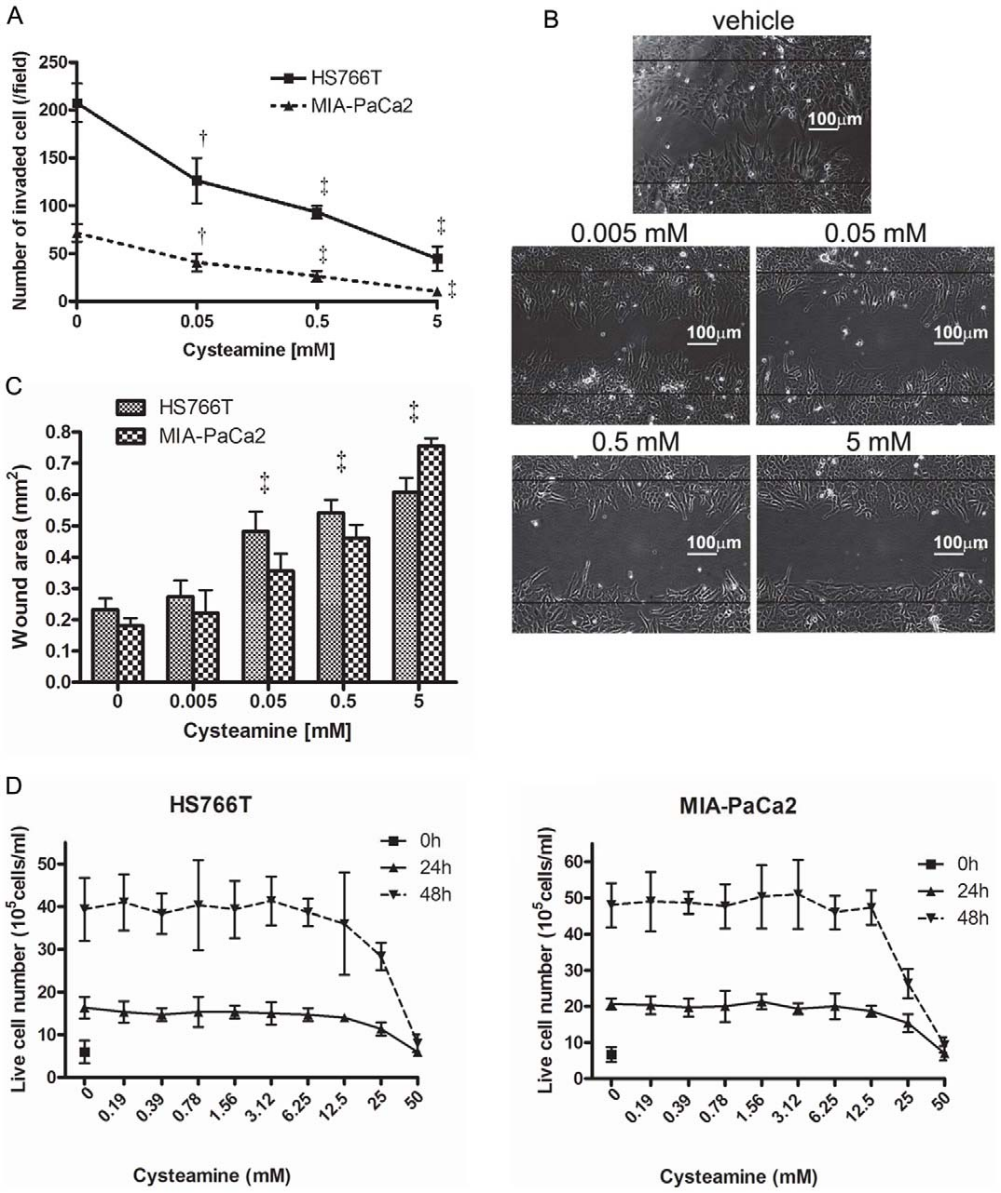
Cysteamine inhibited cell migration and invasion of pancreatic cancer cell lines. A. For the matrigel invasion assay, cells were incubated with increasing concentrations of cysteamine in the matrigel chamber for 24 hours. The number of invaded cells on the opposite side of the membrane was counted. B and C. Wound healing assay. Cells were cultured until confluent and scratched using a sterilized yellow tip. They were incubated for 24 hours with 0–5 mM of cysteamine and the area of the wound between cell layers was measured. The black horizontal lines in figure (vehicle) depict the area at time 0 when wound was made and indicate the area of the wound when cells were scratched (B). D. For cell viability assay, pancreatic cancer cells were incubated with various concentrations of cysteamine and live cell number was counted at 24 and 48 hours. Data are expressed as mean ± S.D. of triplicate determinations. Statistical significances are shown by †: P<0.01 and ‡: P<0.001.

Pancreatic cancer is characterized by local invasion of adjacent structures and metastasis to lymph nodes and liver in the very early stages. At the time of diagnosis, most pancreatic cancers are already metastatic, making the tumor unresectable [15], [16]. Therefore, efforts must be focused on not only targeting the primary tumor but also controlling metastases to successfully treat pancreatic cancer.

Matrix metalloproteinases (MMPs) are a group of zinc-dependent endopeptidases implicated in mammalian angiogenesis, wound healing, and tissue remodeling [17]. In cancer, MMPs play an important role in cell invasion and metastasis by controlling degradation of the extracellular matrix [18]. In particular, MMP-9 plays a central role in pancreatic cancer invasion, and its inhibition decreases liver metastasis of pancreatic cancer [15], [19]. Hence, many MMP inhibitors of broad to narrow specificity have been investigated for their anti-cancer effects. Some MMP inhibitors successfully suppress tumor growth and metastasis in animal models [20]–[22], but they fail to show anti-cancer effects in clinical trials. One reason is their serious adverse effects, particularly those involving severe joint and muscle pain [23], [24]. Therefore, it is important to identify MMP inhibitors with little or no side effects.

**Figure 2:**
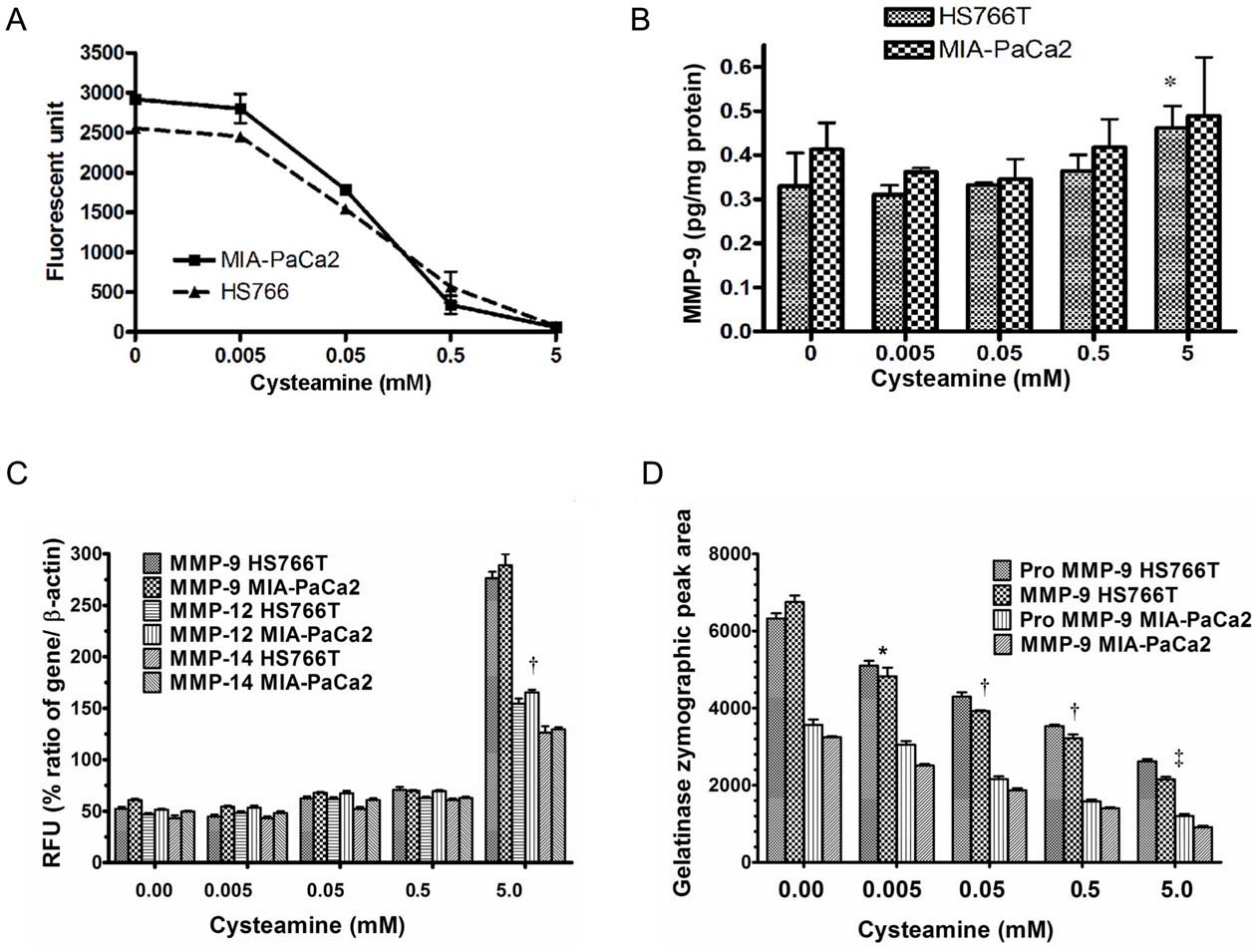
Cysteamine inhibited MMP activity in pancreatic cancer cells. A. To measure total MMPs activity in pancreatic cancer cell lines, cells were incubated with 0–5 mM of cysteamine for 24 hours, total protein extracted and the activity measured using fluorogenic substrate. The effect of cysteamine on MMP-9 at protein level by ELISA (B), MMP-9, MMP-12 and MMP-14 mRNA levels (C) and gelatinase zymographic activities (D) was determined after incubating pancreatic cancer cells with cysteamine for 24 hours. MMP-9 activity was determined by ELISA and mRNA by qRT-PCR. β-actin was used for a reference gene in qRT-PCR. Data represent MMP-9 protein in pg/μg total cell lysate and RFU percent ratio of mRNA/ β -actin. Data are expressed as mean ± S.D. of triplicate determinations and experiments were repeated three times. Statistical significances are shown by *: P<0.05, †: p<0.01 and ‡: P<0.001.

**Figure 3:**
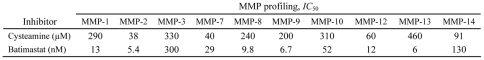
Cysteamine inhibited MMP enzyme activities *in vitro*. *IC*_50_ of MMPs was determined by incubating each MMP enzyme with various concentrations of cysteamine and batimastat in a fluorimetric assay as described in materials and methods. *IC*_50_ is the concentration of inhibitor at which 50% inhibition of proteolysis occurs.

In the present study, we examined the anti-invasive effects of cysteamine, which has an excellent safety profile, in pancreatic cancer cell lines *in vitro*. We also investigated the anti-metastastic- effect of cysteamine in animal models of human pancreatic cancer, specifically determining if it can decrease cancer metastasis in orthotopic pancreatic cancer models in immunodeficient mice. Finally, the safety of subcutaneous cysteamine was assessed in tumor-bearing mice.

## Materials and Methods

### Cell culture and reagents

Pancreatic cancer cell lines (HS766T, MIA-PaCa2, Panc-1, ASPC-1, PK-1, Mpanc96, BxPC-3, KLM, HPAF-II, and SW1990) were obtained from the American Type Culture Collection (Manassas, VA). Cysteamine hydrochloride was purchased from Sigma-Aldrich (St. Louis, MO) and dissolved in distilled water.

### Cell Migration Assay

Cell migration assay is a classical wound healing assay [25]. For this assay, cells were cultured in 10 cm petri dishes until they were completely confluent. Cell monolayers were scraped with a sterile yellow micropipette tip and washed with PBS three times; the cells were then cultured in medium containing 0–5 mM of cysteamine for 24 hours. Five random fields were selected and pictures were taken using an inverted microscope. The average area (mm^2^) of the gap between the cell layers was calculated using IPLab imaging software (BD Biosciences-Bioimaging, Rockville, MD).

**Figure 4:**
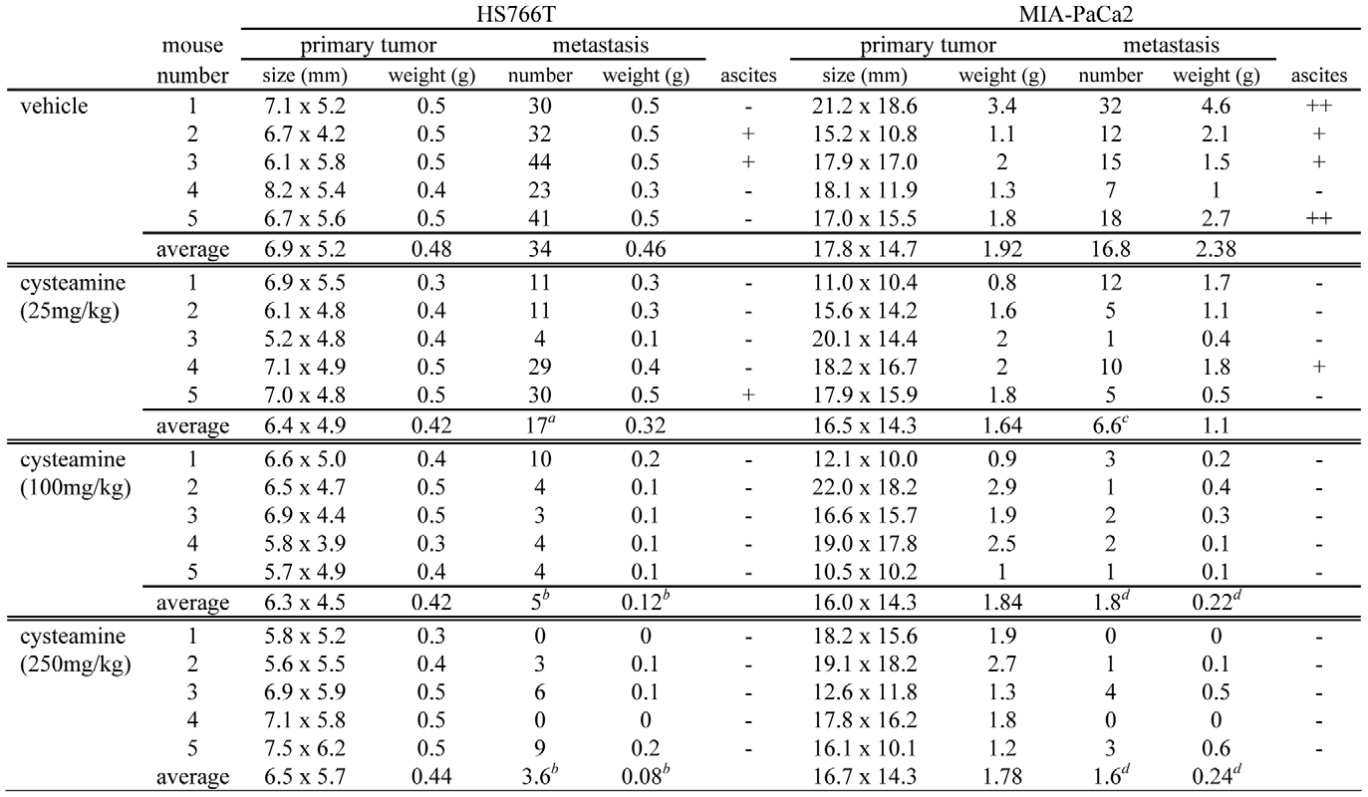
Cysteamine inhibited primary tumors and metastatic lesions in an orthotopic pancreatic cancer *in vivo*. HS766T and MIA-PaCa2 cells (2×10^6^) were implanted directly into the pancreas of 5–6 week-old female nude *nu/nu* mice and then peritoneal cavity and skin were closed using clips. Increasing doses of cysteamine were injected s.c. as described in materials and methods. Primary tumors and metastatic lesions of ≥ 5 mm were counted in vehicle and cysteamine treated mice. *a*: P<0.05, *b*: P<0.01 and *c*: P<0.05, *d:* P<0.001. +: moderate ascites, ++: severe ascites, –: no ascites.

**Figure 5:**
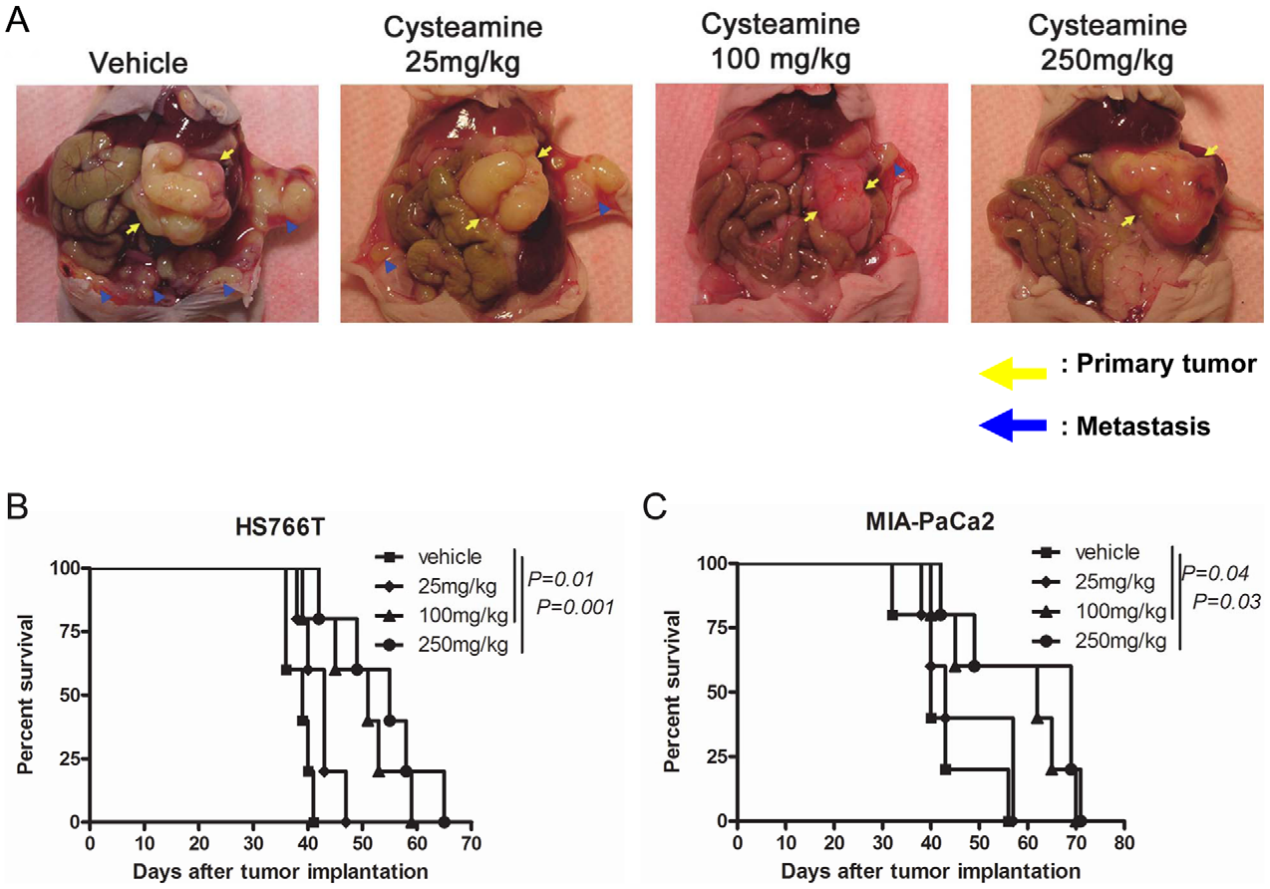
Cysteamine decreased metastasis and prolonged survival in an orthotopic pancreatic cancer mouse model. HS766T and MIA-PaCa2 cells (2×10^6^) were implanted directly into the pancreas of 5–6 week-old female nude *nu/nu* mice and then peritoneal cavity and skin were closed using clips. From day 4 after tumor implantation, mice were treated twice a day with vehicle or 25, 100, 250 mg/kg/day of cysteamine until the end of the experiment. The mice with tumors were sacrificed 4 weeks after cell implantation. A. A representative mouse from each treatment group harboring MIA-PaCa2 tumor is shown. Yellow arrows indicate primary tumors in the pancreas and blue triangles indicate metastatic lesions. In another experiment, control and cysteamine treated mice were followed for survival. Kaplan-Meier survival curves of mice harboring HS766T (B) and MIA-PaCa2 (C) tumors.

### Matrigel invasion assay

Cell invasion was assayed in BD BioCoat Matrigel invasion chambers (BD Biosciences; 24 wells, 8 µm pore size) as described previously [26]. Briefly, cells were incubated with different concentrations of cysteamine (0–5 mM) for 24 hours. Non-invaded cells were removed from the upper surface of the membrane with a cotton swab, and cells on the lower surface of the membrane were fixed and stained with H&E. Three random fields per chamber were counted. Data were shown as mean ± S.D. of triplicate determinations.

**Figure 6:**
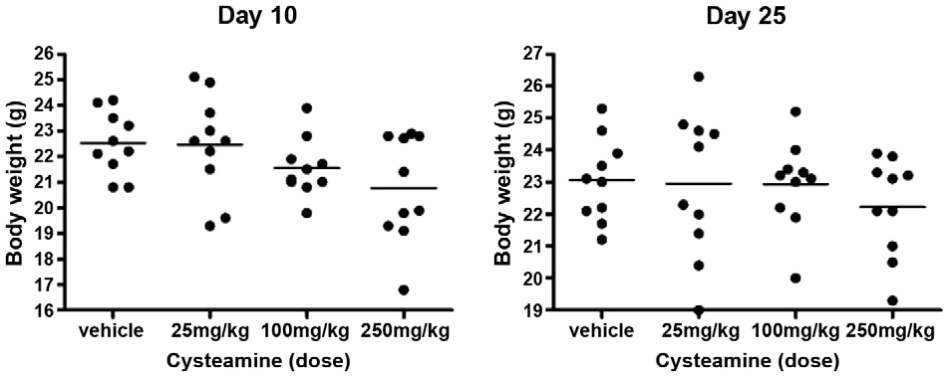
Measurement of body weight of control and cysteamine treated mice. Mice with HS766T tumors were treated with increasing doses of cysteamine and body weights were measured at day 10 and day 25 after tumor implantation. There was no significant difference among treatment groups.

**Figure 7:**
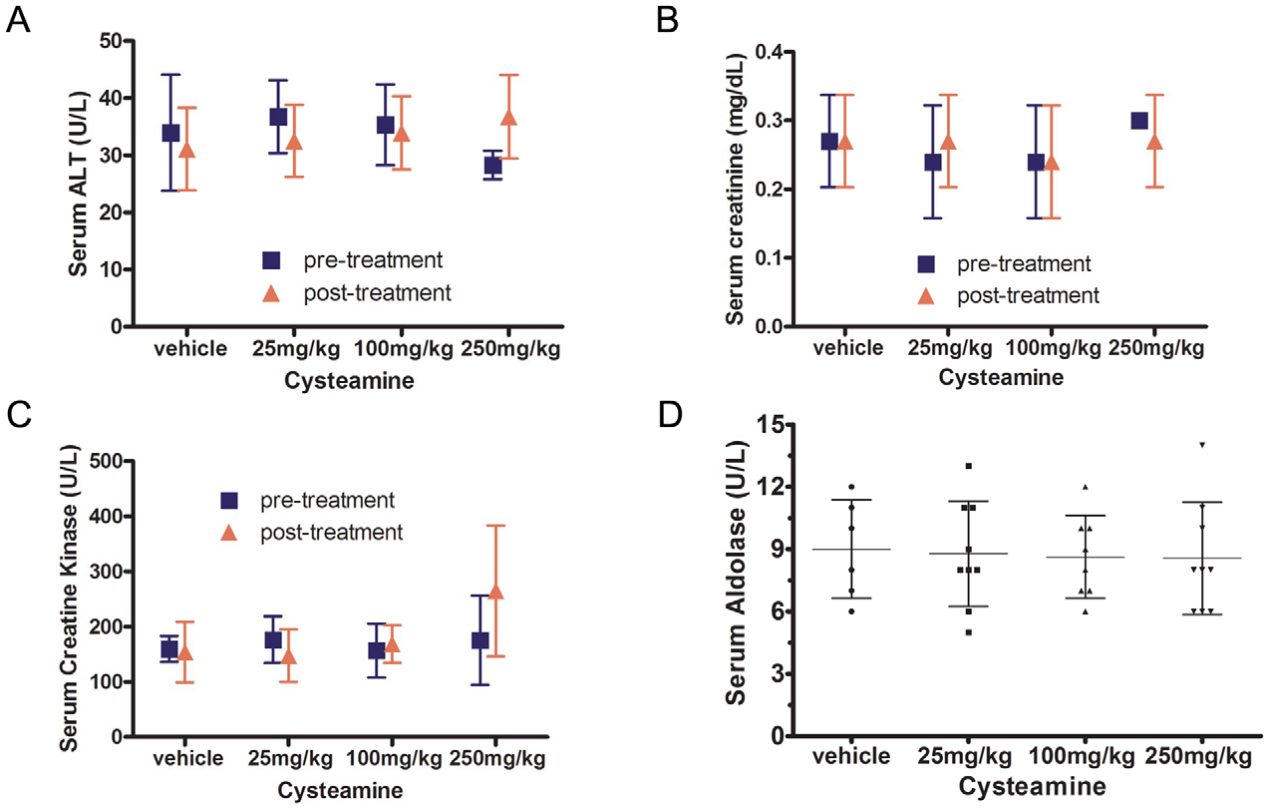
Effect of cysteamine on serum enzymes and creatinine levels. Tumor bearing mice were treated with increasing doses of cysteamine and serum was collected from tail veins twice, i.e., pre-cysteamine treatment (day 4) and post- treatment (day 18). Liver, muscle and kidney function biomarkers were evaluated after cysteamine treatment. Alanine aminotransferase (ALT), creatine kinase (CK), aldolase, and creatinine (Cr) were measured in each group. There were no significant differences among treatment groups.

### Cell viability assay

Cell viability of pancreatic cancer cell lines was measured by counting viable cell numbers. Briefly, 3×10^5^ cells were seeded per well in a 6-well plate with 2.0 milliliter complete medium and incubated overnight for plating. The cells were treated with different concentrations of cysteamine for 24–48 hours. After incubation, cells were detached with trypsin, washed and stained with 0.4% trypan blue. Cell number was manually counted using a hemocytometer and presented as number of viable cells per milliliter.

**Figure 8:**
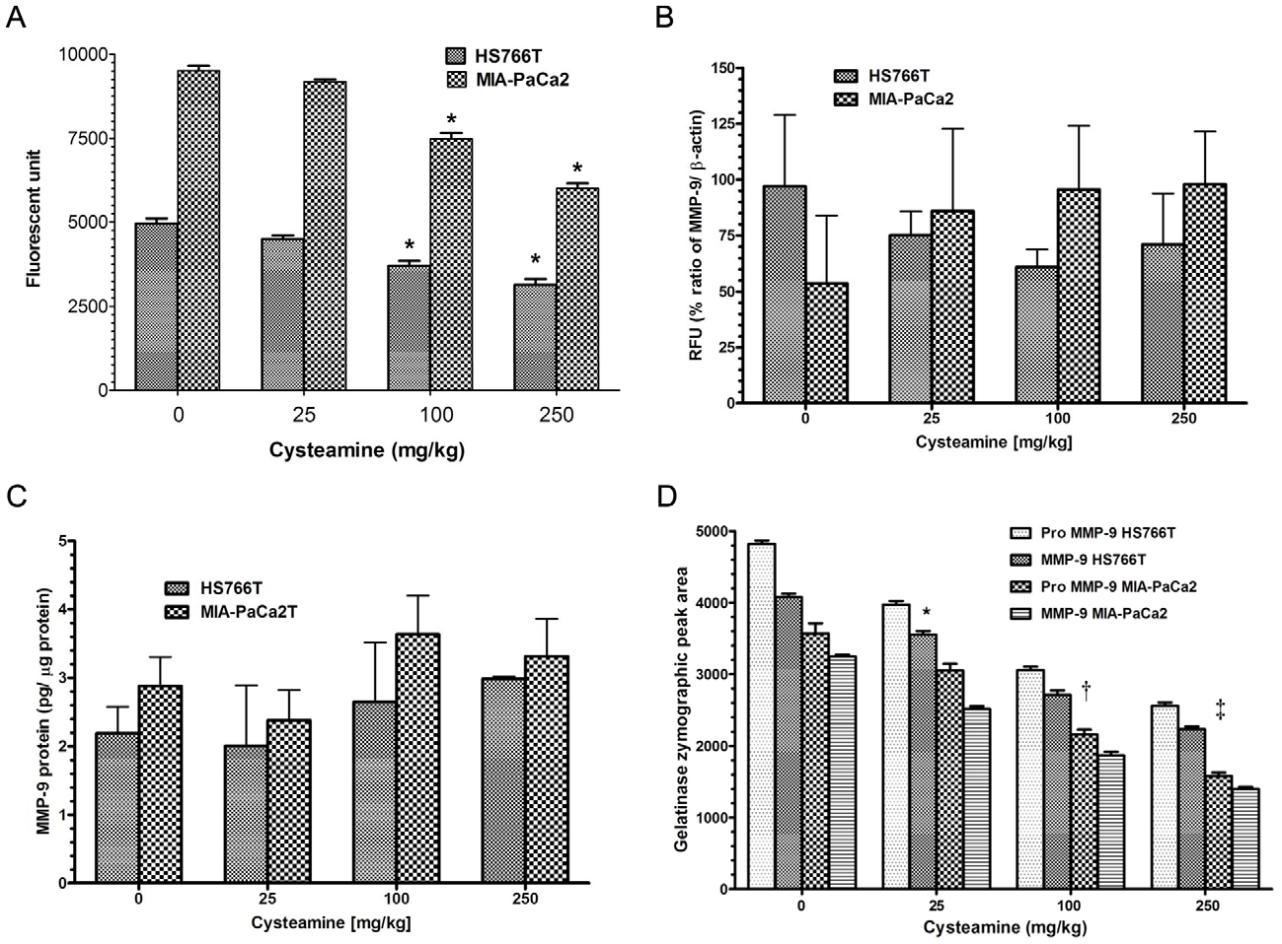
Cysteamine decreased MMP activity in primary orthotopic tumors in mice. A. At day 30 after orthotopic implantation of pancreatic cancer cells, tumors were collected, total protein extracted and MMP activity determined by using fluorogenic MMP substrate. B. MMP-9 mRNA levels were determined in total RNA extracted from tumors by qRT-PCR. β-actin was used for a reference gene. C. MMP-9 protein level was determined by ELISA. D. Zymographic assay was performed in cysteamine treated tumors. Data are expressed as mean ± S.D. of triplicate to quadruplicate determinations. Experiment was repeated two times. Statistical significances are shown by *: P<0.05, †: p<0.01 and ‡: P<0.001.

### Measurement of MMP activity

MMPs activity was measured by Mca-KPLGL-Dpa-AR-NH2 Fluorogenic Peptide Substrate (R&D systems, Emeryville, CA). Total cell protein from each pancreatic cancer cell line was collected using lysis buffer (containing 50 mM Tris-HCl, 10 mM CaCl_2_, 0.05% Brij35 and 0.25% Triton-X) without an MMP inhibitor. Substrate and 10 µg of total cell protein in the buffer were mixed in a black-wall 96-well plate. After 1 hour incubation, fluorescence units were determined using excitation/emission = 320/405 nm.

### Substrate gel zymographic assay for MMP activity

Gelatin gel zymography was performed on cell lysates from HS7666 and MIA-PaCa2 pancreatic cancer cell line with or without culture with different concentration of cysteamine essentially as described by D'Angelo et al [27]. Briefly, 50 µg of total protein was electrophoresed on 10% SDS-PAGE containing 0.1% gelatin as substrate. After washing with 1% Triton X-100 in 50 mM Tris/HCl, pH 7.5 for 1 hr to remove SDS, the gels were incubated overnight at 37^°^C in 50 mM Tris/HCl, pH 7.5, containing 150 mM NaCl, 10 mM CaCl2 and 0.1% Triton X-100, prior to staining with simply blue safe stain. The gels were washed with deionized water at room temperature. Gelatin-degrading enzymes were identified by their ability to digest gelatin as demonstrated by clear zones of digested gelatin. Relevant band intensities were quantified by scanning densitometric analysis and normalized to cell number.

### Quantitative Reverse transcription-PCR

Quantitative reverse transcription-PCR (qRT-PCR) was performed as described previously using QuantiTect SYBR Green PCR Kits (QIAGEN, Valencia, CA) [28]. Gene specific primers for human MMP-9, MMP-12, MMP-14 and β-actin were either purchased from QIAGEN or synthesized at the CBER core facility. Gene expression was normalized to β-actin before the fold change in gene expression was determined.

### Enzyme-Linked ImmunoSorbent Assay (ELISA)

The MMP-9 protein level was determined using Human total MMP-9 DuoSet kit (R&D systems, Emeryville, CA) following the manufacturer's instruction. Ten microgram of total protein was aliquoted in each well and the MMP-9 concentration was determined by the colorimetric method.

### *IC*_50_ of cysteamine against MMPs activity

*IC*_50_ of cysteamine and batimastat was determined using a fluorimetric MMP inhibitor profiling kit (Enzo Life Science, Farmingdale, NY), following the manufacturer's instruction. Briefly, each MMP enzyme was mixed with various concentrations of cysteamine and batimastat in a black-wall 96 well plate and incubated for 30 minutes. After administration of fluorogenic substrate, a velocity of fluorescent increase was measured using excitation/emission = 320/405 nm.

### Measurement of metastasis, survival and body weight of animals implanted with orthotopic pancreatic cancer in mouse model

Female nude *nu/nu* mice between ages 5 and 6 weeks were maintained in a barrier facility in HEPA-filtered rack. All animal studies were conducted under approved protocol #2000–06 by the CBER Institutional Animal Care and Use Committee in accordance with the principles and procedures outlined in *the NIH Guideline for the Care and Use of Laboratory Animals*. For orthotopic tumor cell injection, the pancreas was carefully exposed, and 2.0×10^6^ of MIA-PaCa2 and HS766T cells were injected into the organ. The pancreas was then returned to the peritoneal cavity, and the abdominal wall and skin were closed with skin clips [29],[30]. From day 4 after tumor implantation, increasing doses of cysteamine (0, 25, 100, or 250 mg/kg/day) were subcutaneously injected twice a day until the end of the experiment. At day 30, mice were sacrificed and the number of metastatic nodules visible by naked eye and whose sizes were >5 mm in diameter counted. The total weight of primary tumor and metastatic nodules were also measured. Pictures were taken immediately after sacrificing the animals. Additionally, mouse survival time was monitored in an independent experiment. Mice were sacrificed when they had severe ascites or cachexia. Mice in both tumor models were weighed at day 10 and day 25 after Cysteamine treatment.

### Measurement of enzymes in mouse serum

Mouse blood was collected from the tail vein. Serum levels of alanine aminotransferase (ALT), creatine kinase (CK), aldolase and creatinine (Cr) were measured using different kits obtained from Pointe Scientific, Inc. (Canton, MI) and Caldon Biotech, Inc. (Vista, Ca), following the manufacturer's instruction.

### MMP activity and MMP-9 expression in primary tumors

When mice were sacrificed at day 30, primary tumors were collected. For extraction of total protein, the tumor was soaked with lysis buffer and homogenized using TissueRuptor (QIAGEN); the supernatant was collected. Total RNA was extracted using FastRNA pro green kit (MP Biomedicals, Solon, OH) following the manufacturer's instructions. MMP activity, gelatin hydrolyzing MMP activity and the mRNA and protein levels of MMP-9 were determined as mentioned above.

### Statistical analysis

The data for enzymatic activity, ELISA, and qRT-PCR were compared between each group by ANOVA. Survival curves were generated by the Kaplan-Meier method and compared by using the log-rank test.

## Results

### Cysteamine inhibits pancreatic cancer cell migration and invasion without being cytotoxic to cells

We first examined the effect of cysteamine on pancreatic cancer cell invasion using the matrigel invasion assay. For this assay, we used 10 different pancreatic cancer cell lines in a matrigel invasion chamber. As shown in Fig. 1A and Figure S1, cysteamine inhibited cell invasion in a concentration dependent manner. Even the lowest concentration of cysteamine (0.05 mM) significantly inhibited cell invasion in all pancreatic cancer cell lines.

We next examined the effect of cysteamine on migration of same 10 pancreatic cancer cell lines in the invasion assay. For the migration assay, a wound healing assay was used. The representative pictures of cellular wound healing in HS766T cells are shown in Figure 1B. Cell migration was significantly inhibited at and above 0.05 mM of cysteamine in all 10 cell lines (Figure 1C and Figure S2). The black horizontal lines in figure 1B (vehicle) depict the area at time 0 when wound was made. Since cysteamine mediated similar effects in all 10 cell lines in these assays, we choose two random cell lines for all other assays.

We also examined the cytotoxicity of cysteamine against two pancreatic cancer cell lines (HS766T and MIA-PaCa2). Number of viable cells after 24 and 48 hour cysteamine treatment were counted. Cysteamine showed cell toxicity at >12.5 mM concentration in both cell lines tested but no evidence of toxicity was observed at lower concentrations as number of viable cells were similar to untreated cells (Figure 1D). These results suggest that both cell migration and invasion were inhibited at a noncytotoxic concentration of cysteamine.

### Cysteamine inhibits MMP enzymatic activity in pancreatic cancer cell lines

To investigate the mechanism of inhibition of cell migration and invasion by cysteamine, we examined the effect of cysteamine on MMP enzymatic activity. Cysteamine inhibited MMP activity in two representative pancreatic cancer cell lines tested in a concentration-dependent manner (Figure 2A). The *IC*_50_ {the concentration of cysteamine at which 50% MMP enzyme activity (proteolysis) is inhibited} was calculated by ELISA (Figure 3). Cysteamine directly inhibited each MMP enzymatic activity, with an *IC*_50_ of 38–460 µM. Since MMP-9 plays a central role in pancreatic cancer invasion, we examined its mRNA and protein levels in both cell lines. In contrast to the enzymatic activity, protein and mRNA levels of MMP-9 were modestly increased at the highest concentration of cysteamine (5 mM) (Figure 2B and 2C). We also observed that mRNAs for two other MMPs (MMP-12 and MMP-14) showed a modest increase similar to MMP-9 in response to cysteamine treatment (Fig. 2C). In contrast, zymographic gelatin hydrolyzing activity results for MMP-9 differed significantly from mRNA expression and both pro and active MMP-9 activities decreased significantly in a dose dependent manner (Fig 2D).

### Cysteamine decreases metastasis and prolongs survival of immunodeficient mice implanted orthotopically with human pancreatic cancer

We investigated the anti-metastasis effect of cysteamine in two orthotopic mouse models using human pancreatic cancer cell lines. Mice were treated twice daily with cysteamine subcutaneously from day 4 after tumor implantation until the end of the experiment. At day 30, the number and weight of the primary tumor and metastatic nodules were measured (Figure 4). In both tumor models, the size and weight of the primary tumors did not show any difference between control and treated groups. However, cysteamine significantly decreased the number of metastatic nodules in a dose-dependent manner. At the highest dose (250 mg/kg/day), cysteamine significantly decreased the number of metastases of HS766T tumor by ∼90% (from 34 to 3.6). The total weight of metastatic nodules of MIA-PaCa2 tumor was also decreased by ∼90% at the highest dose. In addition, two of 5 mice in the HS766T tumor model and 4 of 5 mice in MIA-PaCa2 tumor model developed ascites in their peritoneal cavity. However, no mice developed ascites at 100 and 250 mg/kg/day dose in either tumor model. A representative picture of four mice with the MIA-PaCa2 tumor is shown in Figure 5A. We also compared the survival of mice among different treatment groups. Mouse survival time was significantly prolonged when treated with ≥100 mg/kg/day cysteamine in both tumor models (Figure 5B and 5C).

We also carefully monitored the general condition and body weight of mice throughout the experimental period. There was no significant difference in general appearance and body weight among four groups of animals in both tumor models (Figure 6). Similarly, there was no alteration in serum enzymes representing liver function (ALT) or muscle damage (Aldolase) nor evidence of skeletal muscle damage or kidney function (creatinine kinase and creatinine) in the cysteamine-treated groups (Figure 7). Moreover, no organ toxicity was detected in any vital organ such as the liver, kidney, brain, heart, and lung in cysteamine-treated mice when evaluated by histological examination (Figure S3).

### Cysteamine decreases MMP activity in primary orthotopic tumors

MMP activity in primary orthotopic tumors was measured at day 30. Cysteamine decreased MMPs activity in both tumors (HS766T and MIA-PaCa2) at 100 and 250 mg/Kg cysteamine doses (Figure 8A). In the same samples, we measured mRNA by q-RT-PCR and protein levels of MMP-9 by ELISA. In contrast to *in vitro* results, cysteamine did not affect mRNA and protein levels of MMP-9 in primary tumors harvested from mice (Figure 8B and 8C). However, zymography assay for MMP-9 showed a dose dependent decrease in gelatinase activity (Fig. 8D).

## Discussion

We demonstrate that cysteamine inhibits pancreatic cancer cell migration and invasion through direct inhibition of MMP enzymatic activity *in vitro*. This newly discovered property of cysteamine resulted in inhibition of metastasis of human pancreatic cancer cells orthotopically implanted onto pancreas of immunodeficient mice; the effect was cysteamine dose-dependent. Cysteamine decreased not only the number of metastasis in the peritoneal cavity, but also decreased the ascites generated by aggressive pancreatic tumor metastasis. In contrast, no significant change in the size or weight of the primary pancreatic tumor was observed. Consistent with this observation, cysteamine did not cause any effect on cell viability in pancreatic cancer lines up to a concentration which caused significant inhibition of cell migration and invasion. Mice treated with cysteamine survived longer compared to control mice treated with excipient. Both *in vitro* and in primary tumors *in vivo*, MMP enzymatic activity decreased with cysteamine treatment while their expression at mRNA and protein levels did not change. Zymographic results confirmed cysteamine induced MMP inhibition *in vitro* and *in vivo.* These observations indicate that blocking of catalytic activities of MMPs by cysteamine is critical in the inhibition of tumor metastasis in animal model of pancreatic cancer.

The anti-metastatic effects of cysteamine were mediated without any visible signs of toxicity. Mice treated with even the highest dose of cysteamine (250 mg/kg/day) displayed no adverse effects related to general appearance, body weight, muscle damage, serum enzymes and serum creatinine levels. In addition, major organs from treated animals showed no evidence of histological damage. These observations are consistent with the known safety profile of cysteamine in humans [2], [3]. Cysteamine caused only gastrointestinal symptoms in subjects, as it increased gastric acid production and decreased gastrointestinal motility [31]. However, these effects were controlled by concomitant use of proton-pump inhibitor [32]. It is noteworthy that cysteamine sufficiently inhibited cancer cell migration and invasion *in vitro* at 50 µM, a concentration that can be achieved *in vivo*. Oral administration of cysteamine (given every 6 hours at 60 to 90 mg/kg of body weight per day) can increase the cysteamine plasma level up to ∼50 µM [3], [33]–[35]. In addition, 100 mg/kg/day s.c. injection of cysteamine in animals is similar to the dosage used for cystinosis, and this dose produced a significant decrease in tumor metastasis and prolongation of survival. We consider that cysteamine may be safely administered in the clinic to control pancreatic cancer metastasis.

Both mRNA and protein levels for MMP-9 were modestly to moderately upregulated *in vitro* at the highest dose of cysteamine. Similarly, mRNA of MMP-12 and 14 were also modestly upregulated at the highest dose. This increase in MMP-9 was not observed *in vivo*, perhaps because the pancreatic cancer cells were incubated with cysteamine only for 24 hours, while *in vivo* tumors were exposed to cysteamine continuously for 27 days. We did not attempt to measure cysteamine levels in tumors *in vivo* because intracellular metabolic fate of cysteamine *in vivo* is very complex and difficult to measure as it binds to free thiol, particularly the cysteines of cellular proteins. Instead, we depended mainly on the biological effects of cysteamine on MMP activities and tumor growth. Nevertheless, the increased MMP-9, MMP-12 and MMP-14 levels at the highest concentration *in vitro* may represent a temporary compensatory effect of cells due to a sudden decrease of MMP activity by cysteamine. In contrast, the catalytic activity of MMP-9 to hydrolyze gelatin in zymography assay did not show such upregulation after treatment with highest dose of cysteamine of pancreatic cancer cell lines *in vitro* as well as orthotopic tumors *in vivo*. In fact in zymography assay for MMP-9, cysteamine caused a dose dependent decrease in gelatinase activity. These results suggest that enzymatic inhibition of MMP-9 by cysteamine may be involved in decrease of invasion and metastasis of pancreatic cancer. MMP1, MMP2, MMP7, and MMP9 have also been shown to be expressed in pancreatic tumor [36], [37], but the effect of cysteamine on protein and mRNA levels for all these MMPs was not examined except for MMP9. Thus, present study lacks data on the effect of cysteamine on protein and mRNA levels for all MMPs including MMP2. However, it is important to point out that cysteamine inhibited enzymatic activity of MMPs, which includes all MMPs.

The anti-MMP activity of cysteamine was lower than that of specific MMP inhibitors such as batimastat and marimastat (*IC_50_* nM to low μM compared to high μM range for cysteamine), but cysteamine may be better tolerated *in vivo*. In fact, batimastat had problems of poor oral bioavailability [24] and marimastat failed in the clinic as higher dosage produced high musculoskeletal toxicities and poor survival in patients with breast cancer metastasis [38]. In the present study, mice tolerated up to 250 mg/kg/day cysteamine without any visible, biochemical or histological evidence of toxicity or muscular damage.

In previous cancer treatment studies, cysteamine was used because of its anti-oxidative and radio-protective effects [39]. During these studies, it was observed that cysteamine also had anti-carcinogenic and anti-proliferative activities in a variety of cancers. We now report that cysteamine exerts anti-MMP and anti-metastatic effects. However, cysteamine did not affect the primary pancreatic tumor size, suggesting that it should be used concurrently with other anti-tumor agents such as gemcitabine for optimal anticancer effects [13]. Based on these insights, we propose that cysteamine can be useful as mono-therapy prior to surgery to prevent metastasis, as an adjuvant, or as a component of combination therapy for advanced stage disease to prolong survival of patients with pancreatic cancer.

## Supporting Information

Figure S1 **Cysteamine inhibited cell invasion of pancreatic cancer cell lines.** For the matrigel invasion assay, cells were incubated with increasing concentrations of cysteamine in the matrigel chamber for 24 hours, as for Figure 1A. The number of invaded cells on the opposite side of the membrane was counted. The average number was calculated in three individual fields per the chamber and the data are expressed as mean ± S.D. of triplicate determinations.
(TIF)

Figure S2 **Cysteamine inhibited cell migration of pancreatic cancer cell lines.** For the wound healing assay, cells were cultured until confluent and scratched using a sterilized yellow tip. They were incubated for 24 hours with 0–5 mM of cysteamine and the area of the wound between cell layers was measured. Data are expressed as mean ± S.D. of five different-field determinations.
(TIF)

Figure S3 **Histological analysis of vital organs in cysteamine and control treated mice.** Tumor-bearing immunodeficient mice were treated with increasing doses of cysteamine and vital organs (liver, kidney, brain, heart, and lung) were harvested from each group at day 30 after treatment. Tissue sections were analyzed by hematoxylin and eosin staining. No organ toxicity by cysteamine was detected.
(TIF)
