# Supplementary figures and images for: Cysteamine Suppresses Invasion, Metastasis and Prolongs Survival by Inhibiting Matrix Metalloproteinases in a Mouse Model of Human Pancreatic Cancer
Source: PLoS One. 2012 Apr 20;7(4):e34437. doi: 10.1371/journal.pone.0034437 (PMC3332081; doi:10.1371/journal.pone.0034437)

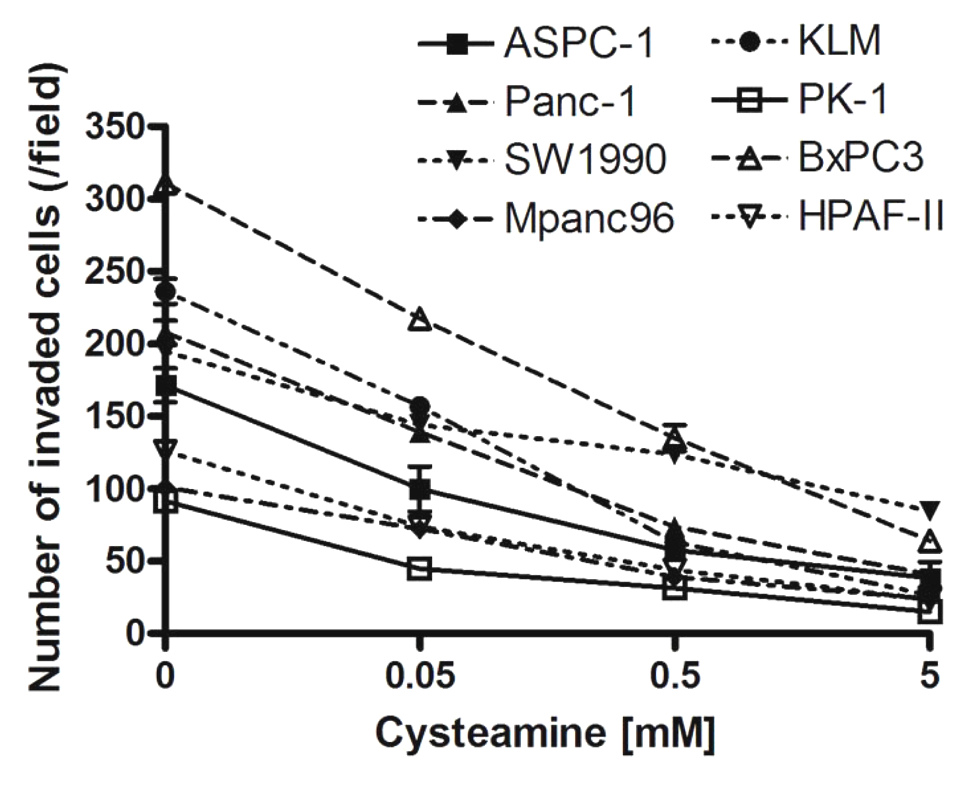

Supplement: Figure S1 — Cysteamine inhibited cell invasion of pancreatic cancer cell lines. For the matrigel invasion assay, cells were incubated with increasing concentrations of cysteamine in the matrigel chamber for 24 hours, as for Figure 1A. The number of invaded cells on the opposite side of the membrane was counted. The average number was calculated in three individual fields per the chamber and the data are expressed as mean ± S.D. of triplicate determinations. (TIF) [file pone.0034437.s001.tif]

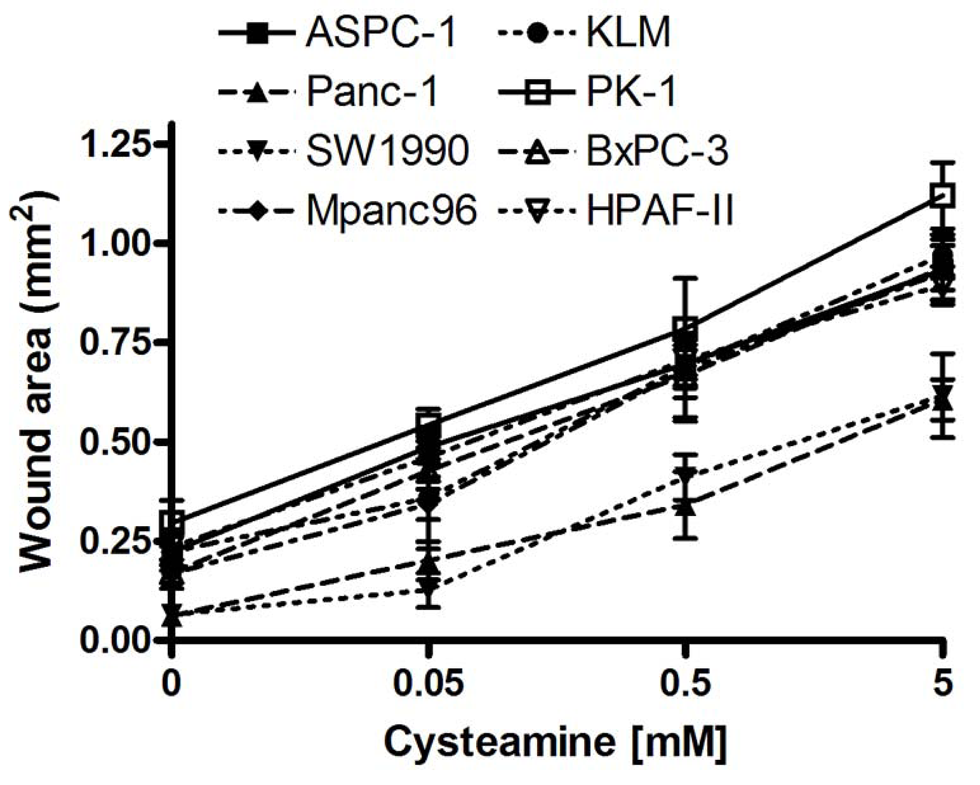

Supplement: Figure S2 — Cysteamine inhibited cell migration of pancreatic cancer cell lines. For the wound healing assay, cells were cultured until confluent and scratched using a sterilized yellow tip. They were incubated for 24 hours with 0–5 mM of cysteamine and the area of the wound between cell layers was measured. Data are expressed as mean ± S.D. of five different-field determinations. (TIF) [file pone.0034437.s002.tif]

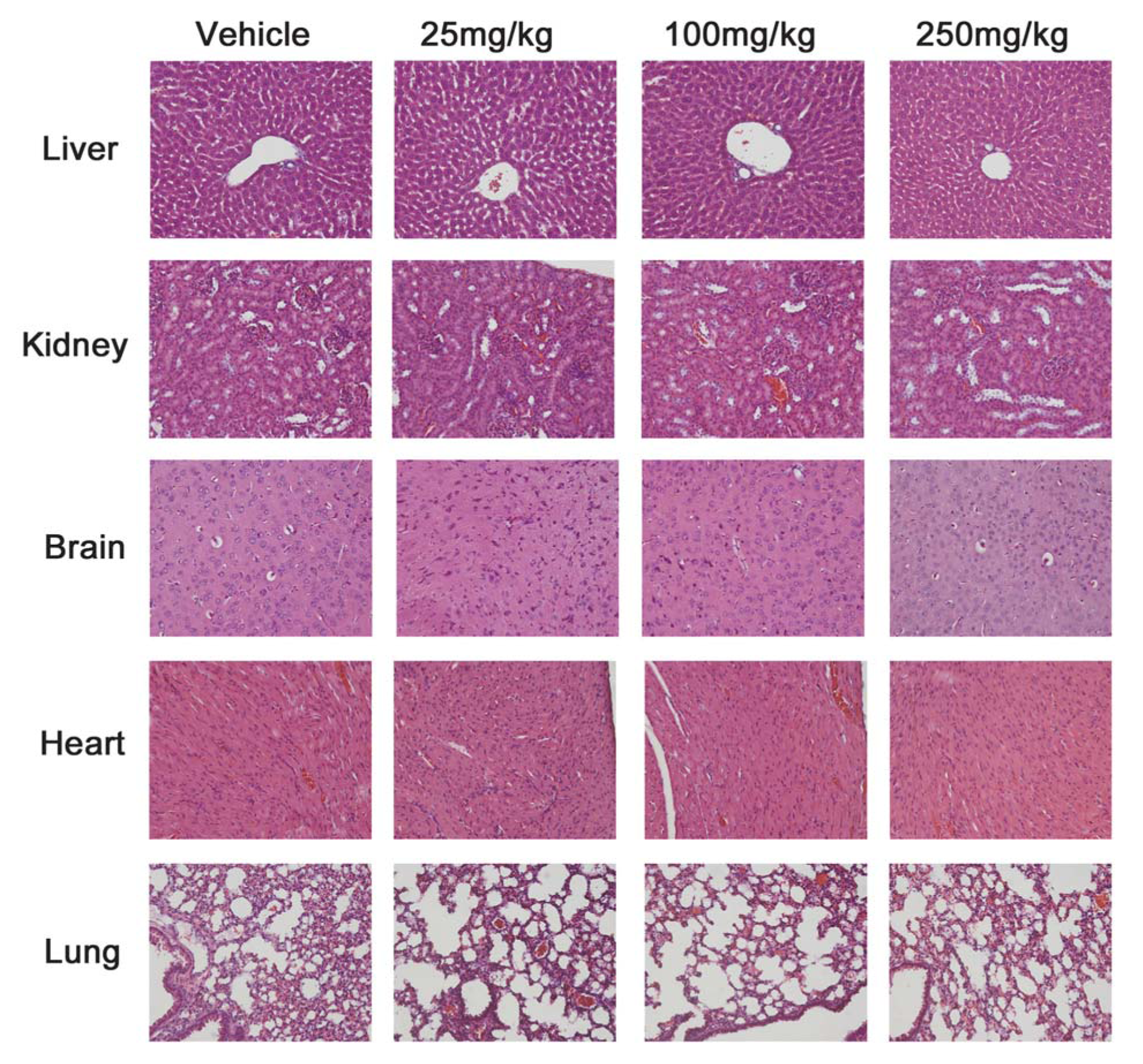

Supplement: Figure S3 — Histological analysis of vital organs in cysteamine and control treated mice. Tumor-bearing immunodeficient mice were treated with increasing doses of cysteamine and vital organs (liver, kidney, brain, heart, and lung) were harvested from each group at day 30 after treatment. Tissue sections were analyzed by hematoxylin and eosin staining. No organ toxicity by cysteamine was detected. (TIF) [file pone.0034437.s003.tif]
